# First isolation and genotyping of pathogenic *Leptospira* spp. from Austria

**DOI:** 10.1038/s41598-024-53775-w

**Published:** 2024-02-26

**Authors:** Cynthia Sohm, Denise Willixhofer, Eva Fasching, Karoline Waldner, Nicole Deitzer, Janina Steiner, Julia Jöbstl, Corina Schleicher, Marcel Schwarz, Reinhard Fuchs, Pascale Bourhy, Annemarie Käsbohrer, Thomas Wittek, Clair Firth, Romana Steinparzer, Amélie Desvars-Larrive

**Affiliations:** 1 VetFarm, Department of Farm Animals and Veterinary Public Health, University of Veterinary Medicine Vienna, Kremesberg 13, 2563 Pottenstein, Austria; 2 Unit of Veterinary Public Health and Epidemiology, Department of Farm Animals and Veterinary Public Health, University of Veterinary Medicine Vienna, Veterinärplatz 1, 1210 Vienna, Austria; 3 University Clinic for Ruminants, Department of Farm Animals and Veterinary Public Health, University of Veterinary Medicine Vienna, Veterinärplatz 1, 1210 Vienna, Austria; 4 Institute for Veterinary Disease Control, Austrian Agency for Health and Food Safety (AGES), Robert Koch-Gasse 17, 2340 Mödling, Austria; 5 Department for Data, Statistics and Risk Assessment, Austrian Agency for Health and Food Safety (AGES), Zinzendorfgasse 27/1, 8010 Graz, Austria; 6 Unit Biology of Spirochetes, Department of Microbiology, Institut Pasteur, 25-28 Rue du Docteur Roux, 75015 Paris, France; 7 Complexity Science Hub Vienna, Josefstädter Straße 39, 1080 Vienna, Austria

**Keywords:** Bacterial infection, Bacteria

## Abstract

Leptospirosis is a globally distributed zoonotic disease. The standard serological test, known as Microscopic Agglutination Test (MAT), requires the use of live *Leptospira* strains. To enhance its sensitivity and specificity, the usage of locally circulating strains is recommended. However, to date, no local strain is available from Austria. This study aimed to isolate circulating *Leptospira* strains from cattle in Austria to enhance the performances of the routine serological test for both humans and animals. We used a statistical approach combined with a comprehensive literature search to profile cattle with greater risk of leptospirosis infection and implemented a targeted sampling between November 2021 and October 2022. Urine and/or kidney tissue were sampled from 410 cattle considered at higher risk of infection. Samples were inoculated into EMJH-STAFF culture media within 2–6 h and a real-time PCR targeting the *lipL32* gene was used to confirm the presence/absence of pathogenic *Leptospira* in each sample. Isolates were further characterised by core genome multilocus sequence typing (cgMLST). Nine out of 429 samples tested positive by PCR, from which three isolates were successfully cultured and identified as *Leptospira borgpetersenii* serogroup Sejroe serovar Hardjobovis, cgMLST cluster 40. This is the first report on the isolation and genotyping of local zoonotic *Leptospira* in Austria, which holds the potential for a significant improvement in diagnostic performance in the country. Although the local strain was identified as a cattle-adapted serovar, it possesses significant zoonotic implications. Furthermore, this study contributes to a better understanding of the epidemiology of leptospirosis in Europe.

## Introduction

Leptospirosis is a globally occurring zoonotic disease caused by pathogenic bacteria of the genus *Leptospira*. The disease is a growing public health concern worldwide^1^. In Central Europe, annual leptospirosis morbidity and mortality are estimated at 4.02 cases (95% CI 1.21–6.85) and 0.21 deaths (95% CI 0.08–0.33) per 100,000 population, respectively^2^. However, the incidence of the disease is expected to increase as a result of global change, particularly due to climate warming and increasing urbanisation^3^.

In Austria, rats and pigs are considered common carriers of leptospires^4,5^. The first outbreak of human leptospirosis in Austria was reported in 2010 in Langau, Lower Austria, among triathlon participants^6^ and, in 2021, the national incidence (0.17 cases per 100,000 population) laid slightly below the average incidence in EU/EEA countries (0.20)^7^. The disease in humans presents mild (unspecific, influenza-like symptoms) to severe forms, which may lead to icterus and multi-organ failure, specifically a syndrome known as Weil’s disease^8^ that has previously been reported in Austria^9^. Moreover, ten percent of reported human autochthonous cases in Austria are related to farm animals^10^, emphasising the necessity to effectively investigate the livestock-human interface. In particular, bovine leptospirosis has a significant economic impact, as the main symptoms in cattle include abortions, fertility issues, and a loss in milk production^11,12^.

The Microscopic Agglutination Test (MAT) is the gold standard for the serological diagnosis of leptospirosis in both humans and animals. The MAT requires the use of panels of live reference strains, representative of all serogroups, that are incubated with serial dilutions of the patient sera. When antigen–antibody reactions occur, agglutinates can be observed under dark-field microscopy^8^. To achieve optimal sensitivity and specificity of the MAT, the World Health Organization (WHO) and the World Organisation for Animal Health (WOAH) recommend incorporating locally circulating *Leptospira* strains into the testing panel^13,14^. However, the culture and isolation of *Leptospira* are challenging. The bacteria often exhibit slow growth during primary isolation, culture may take several months depending on the serovar^15,16^, and some fastidious strains require specific culture medium formulations^17^. Additionally, the method shows low sensitivity and is therefore rarely used in clinical settings. Yet, prevention and control of leptospirosis rely on the understanding of its local epidemiology, including knowledge on local strains and hosts involved. While sequencing of DNA amplicons obtained from field samples can characterise the infecting *Leptospira* strain to the species level, it does not provide information on the serogroup. Similarly, multilocus sequence typing is a promising tool for studying the sequence polymorphism of *Leptospira* directly from clinical samples^18^. However, this method requires a high concentration of leptospires in the specimen^19^ and is typically applicable only to cultured isolates^20^. Consequently, *Leptospira* isolation remains an essential prerequisite for comprehensive molecular and serological characterisation of the bacteria, e.g., whole genome sequencing, proteomics.

Until now, the sole documented isolate from Austria was obtained from field voles in 1977 and identified as *Leptospira* serovar (sv.) saxkoebing^21^. However, these isolates have not been available and cannot be used for diagnostic purposes, which rely on non-domestic strains. Incorporating a locally circulating strain into the MAT panel used for routine diagnostic will certainly enhance diagnostic capabilities in Austria^13^ and may allow for the identification of additional cases in humans, but also in animals. The objective of this study was to develop and implement a pertinent sampling strategy and culturing method to isolate circulating *Leptospira* strains in Austria. This aimed to enhance the performances of the serological diagnostic for humans and animals in the country and further our understanding of the local epidemiology of this zoonotic disease.

## Materials and methods

### Study design

Sampling was carried out in Austria, specifically, in the north-eastern federal state of Lower Austria, from November 2021 to September 2022. Cattle were chosen over other species for this study due to ease of accessibility, the potential significant economic impact of the disease, and the scarcity of available data in this species, which needed to be addressed.

We aimed to isolate and further maintain in culture ten *Leptospira* strains. We considered a probability of success equal to 0.03, based on a previous study^22^ and used a negative binomial distribution to estimate the required sample size. It was determined that 410 animals were required to achieve our objective with a probability of 80%.

#### Targeted sampling

Pathogenic *Leptospira* persist in renal tissue in their host and are intermittently excreted in the urine of infected animals^8^. To maximise our chance of isolating circulating *Leptospira* strains, we developed a targeted sampling strategy and focused our sampling effort on:

i. Urine collected on farms, from live animals presenting with clinical signs compatible with leptospirosis (as reported by the herd veterinarian or the animal owner);
ii. Kidney tissues and, if possible, urine from routinely slaughtered (i.e., clinically healthy) cattle originating from farms that were considered at high risk of leptospirosis.

Both dairy and beef cattle were considered in the study. Inclusion criteria for animals and farms were established based on (i) a literature review that identified the most frequently reported clinical signs and risk factors of bovine leptospirosis in Europe^23^ and (ii) a retrospective analysis of national bovine leptospirosis serological data (data source: Laboratory Information and Management System database, Austrian Agency for Health and Food Safety). This statistical analysis incorporated serological data from 8431 cattle from 3030 farms in Austria, which were tested for leptospirosis in 2015–2021. In addition, data on the animal age, breed, sex, calving, (community) pasture use, and cattle movements, as well as data on the farm production type and region were acquired from the Austrian Consumer Health Information System (Verbrauchergesundheitsinformationssystem, VIS). A Generalised Linear Mixed Model (GLMM) was then performed to identify risk factors of bovine leptospirosis in Austria. The farms, where the animals were kept at the time of testing, were treated as a random variable. Seropositivity was defined as MAT titres ≥ 100 against at least one pathogenic reference serogroup. Model and variable selection were performed by forward selection using the Bayesian Information Criterion (BIC) and a five-fold cross validation using the Matthews correlation coefficient (detailed description of the methods and results of this analysis is provided in Supplementary Material 1).

#### Semi-targeted sampling

After three months of sampling, the targeted sampling approach did not yield the expected sample size, due to a limited recruitment of farms during that period. Consequently, we implemented, in parallel, a semi-targeted approach consisting of sampling cattle chosen at the slaughterhouse, using the limited information that could be provided by the slaughterhouse, in strict compliance with the General Data Protection Regulation (GDPR). Inclusion criteria, as predefined for the targeted sampling, were used. Because of logistical constraints, only kidney tissue was collected from these animals.

**Figure 1:**
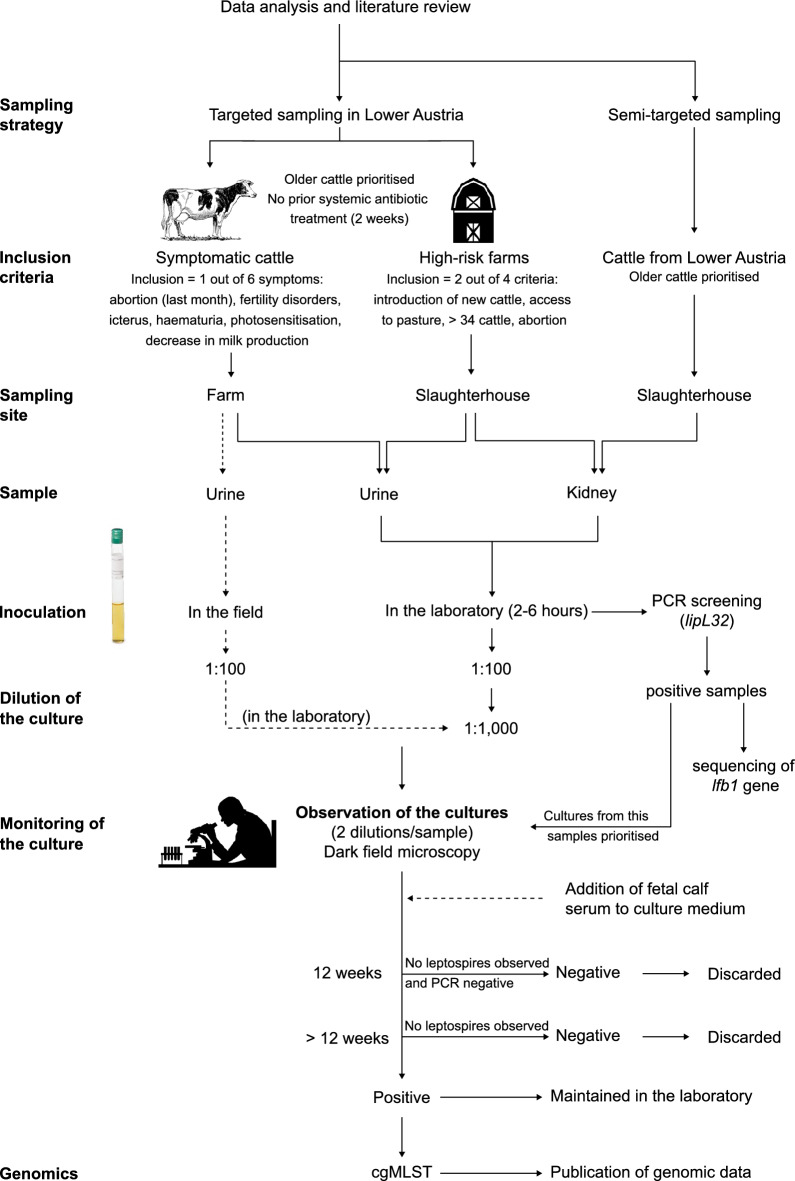
Workflow of the study, from development of the sampling protocol to successful isolation of locally circulating pathogenic *Leptospira*, Austria, November 2021–September 2022.

The sampling strategy and utilisation of samples in the laboratory are depicted in Fig. 1.

### Sample and data collection

Sterile urine was obtained from symptomatic live cattle on farm by catheterisation of the urinary bladder. If the cow urinated immediately prior to sampling, the middle stream of the urine was collected. At slaughterhouses, the urine was punctured from the urinary bladder immediately after it was removed from the carcass. Similarly, kidney tissue was collected immediately after slaughter and removal from the carcass. The kidney surface was cleaned with denatured 70% ethanol and sterile water and one 1 cm^3^ fragment was severed from the corticomedullary junction^24^ using a sterile scalpel blade.

After sampling, each urine and kidney sample were transferred into individual 40 mL sterile containers and kept at room temperature until processing. To optimise the survival and viability of the *Leptospira,* urine and kidney samples were transferred to the laboratory within two to six hours, respectively^22^.

Data on animals sampled within the targeted sampling protocol were obtained from the animal owners via a questionnaire (e.g., age, sex, management practices, and observed clinical signs). Data on animals sampled within the semi-targeted sampling protocol (i.e., federal state of origin and age) were retrieved from the slaughterhouse database.

### Culture of *Leptospira*

Samples were immediately processed upon arrival at the laboratory. One hundred microlitre of urine were directly inoculated into 10 mL Ellinghausen-McCullough-Johnson-Harris (EMJH) culture tube (Difco™, Becton Dickinson, USA) (1:100 dilution) supplemented with the selective medium STAFF (40 µg/mL sulfamethoxazole, 20 µg/mL trimethoprim, 5 µg/mL amphotericin, 200 µg/mL fosfomycin, 100 µg/mL 5-fluorouracil)^25^. Each kidney fragment was placed into 10 mL phosphate buffered solution (PBS), homogenised in a stomacher for 10 min, and 100 µL of the homogenate were transferred into 10 mL of EMJH-STAFF culture medium. For each culture, two serial dilutions were prepared, 1:100 and 1:1000, with the second dilution containing STAFF (prior to May 2022) or not (from May 2022 on). From October 2022, to support the primary growth of leptospires, 1 mL foetal calf serum (FCS) was added to the cultures of PCR-positive samples (1:10 dilution with EMJH), following the protocol of Chideroli et al.^17^. Cultures were incubated at 29 °C and examined weekly under a dark-field microscope for 12 weeks before being discarded. If contaminants were detected, a filtration (PALL^®^ Acrodisc^®^ PF 32 mm Syringe Filter with 0.8/0.2 µm Supor^®^ Membrane) was performed before inoculation into fresh EMJH-STAFF medium, followed by a serial dilution of the culture. Cultures of PCR-positive samples were discarded after 30 weeks if no growth occurred.

Despite the detection of *Leptospira* DNA through PCR screening, initial efforts to isolate the strains from bovine urine samples were unsuccessful. Hence, as from 11 May 2022, to minimise the time gap between sampling and inoculation and therefore enhance the viability of the leptospires, urine samples collected from live symptomatic animals (targeted sampling) were inoculated directly in the field. The mobile laboratory was safely placed to manipulate the samples and avoid potential contamination from the animals (e.g., at least 50 m from the barn, protected from the sun and wind). To achieve a sterile environment, the samples were handled above an alcohol-disinfected, dry surface and within a sterile field created by a Bunsen burner (when meteorological conditions allowed its use). The inoculation at a dilution 1:100 was implemented in the field as described above; the second dilution (1:1000) was performed in the laboratory.

### DNA extraction, PCR screening, and sequencing

Pelleted urine (1.5 mL centrifuged for 20 min 13,000 rpm, followed by a wash step with 1 mL PBS and another centrifugation step) and homogenised kidney tissues (see above) were stored at − 20 °C until further processing. Prior to DNA extraction, urine pellets were diluted with 140 µL PBS while 200 µL homogenised kidney tissues were added to 180 µL buffer ATL and 20 µL proteinase K. DNA was extracted using a commercial kit (BioExtract^®^ SuperBall^®^, BioSellal, France). Real-time PCR was performed using primers targeting the *lipL32* gene^26^. Negative (PCR Grade Water) and positive (DNA from *Leptospira interrogans*) controls were included in each run to monitor amplification and any potential inhibitor. Samples that tested positive for the presence of *Leptospira* by PCR were prioritised for subsequent culture follow-ups. Additionally, to determine the genomic species, the *lfb1* gene amplification products of the PCR-positive samples were sequenced, as described by Garcia-Lopez et al.^27^.

### Characterisation of the isolates

The MAT was used for serogroup characterisation with a standard battery of rabbit antisera against reference serovars representing the 24 serogroups^28^.

Genomic identification of the isolated *Leptospira* species and genovar was performed using a core genome multilocus sequence typing (cgMLST) scheme based on 545 highly conserved loci and clonal groups (CG) were defined using a single linkage clustering threshold of 40 allelic mismatches, as developed by Guglielmini et al.^29^. This method facilitates standardised genomic taxonomy, data sharing, and comparison of *Leptospira* strains (https://bigsdb.pasteur.fr/leptospira).

### Ethics approval

This study followed institutional and national standards for the care and use of animals in research. This study was discussed and approved by the institutional ethics and animal welfare committee of the University of Veterinary Medicine Vienna, Austria, in accordance with good scientific practice guidelines and national legislation (ETK-038/03/2021). Informed written consent of the animal owners and the slaughterhouse managers was obtained before collecting samples.

As the study required the collection of data relating to human subjects (e.g., contact details), animals, and farming practices, it was submitted to the Ethics Committee of the Medical University of Vienna, Austria. This Ethics Committee decided that an official decision on the present study was not required, in accordance with the current Austrian legislation.

## Results

### Characteristics of the animals, farms, and samples

#### Targeted sampling

##### Animals considered at higher risk of leptospirosis

Most reported clinical signs of bovine leptospirosis in Europe, as identified in a literature review were: abortion, fertility disorders, photosensitisation, decrease in milk production, haematuria, and icterus^23^. The targeted sampling focused therefore on animals presenting with at least one of these clinical signs in the last month (as reported by the veterinarian or the animal owner).

Overall, 101 clinically symptomatic animals from 22 farms were sampled as part of the targeted sampling protocol, of which two cows were sampled twice, resulting in 103 urine samples. All symptomatic animals were female, with age ranging 2–13 years (median: 5 years). The main reported clinical signs in sampled animals were fertility disorders (e.g., cow typically requiring two or more inseminations to achieve pregnancy, prolonged calving intervals) (82%) and/or abortions (30%). Some animals presented more than one symptom (epidemiological information on the investigated cattle is available in Supplementary Table 1).

##### Farms considered at higher risk of leptospirosis

Most common risk factors positively associated with leptospirosis in European cattle farms were: access to pasture, external purchase of animals, history of abortion, and herd size^23^. The retrospective analysis of national data showed that 441/8431 animals serologically tested positive for leptospirosis between 2015 and 2021 (5.2%; 95% CI 4.8–5.7). All implemented models showed that variables related to community pasture significantly increased the risk of leptospirosis, with the odds of a positive sample increasing by a factor of 1.22 (p-value = 0.004) per additional stay at a community pasture. Cattle that had not (yet) calved presented a significantly lower risk of testing positive compared to cattle that had already calved (OR = 0.22, p-value < 0.001). Consequently, farms were considered as high-risk for leptospirosis if two or more of the following risk factors were present: access to pastures, history of abortion, animal replacements off-farm, or large herd size. Since the average herd size in Austria is 34 head of cattle^30^, we considered farms with > 34 animals to be larger than average. Moreover, since both the literature review^23^ and statistical analysis indicated that older animals (i.e. that have already calved) had higher odds of being infected than younger ones (p < 0.001, Supplementary Material 1), we prioritised sampling of older cattle. Additionally, only cattle that did not receive a systemic antibiotic treatment within two weeks prior to sampling were included in the study. None of the sampled animals had been vaccinated since vaccination against leptospirosis is not routinely conducted in Austria.

With the support of local veterinarians, ten farms were identified as at higher risk of *Leptospira* infection and included in the targeted sampling protocol (of which four were also visited because they had symptomatic animals). The total number of cattle per farm varied from 22 to 105 animals (median: 62.5). All farms provided the cattle with access to pasture, six of them regularly introduced new cattle, and eight reported abortions within the past two years (epidemiological information on the investigated farms is available in Supplementary Table 2). From these 10 farms, 27 animals were sampled at the slaughterhouse (both kidney tissue and urine were collected from 16 cattle, while solely kidney tissue was collected from a further 11 animals, due to logistical constraints).

#### Semi-targeted sampling

In the framework of the semi-targeted protocol, 283 cattle, originating from Lower Austria, were sampled from five slaughterhouses. Information on age was collected for 235 animals (83%) and ranged from one to 16 years (median: 4 years) (Supplementary Table 1).

#### Samples

A total of 429 samples, from 410 individual animals, were collected. Among them, 146/429 (34%) were obtained in the framework of the targeted sampling. In total, 310 kidney (72.3% of all samples) and 119 urine samples (27.7%) were included in the study. The average time from sample collection to laboratory processing was 3h03 for kidney samples and 2h09 for urine samples (a detailed description of the samples is provided in Supplementary Table 3).

### PCR and sequencing

We evidenced a *Leptospira* carriage prevalence of 1.2% (5/410) among investigated animals (95% CI 0.5–2.8). Specifically, six urine samples, obtained from four animals (two individuals were sampled twice), and three kidney samples, obtained from three animals, tested positive for pathogenic *Leptospira* via *lipL32*-based PCR, with Ct values ranging from 25 to 37 (Table 1). Two animals had positive results for both urine and kidney. All PCR-positive animals were sampled through the targeted strategy and originated from a single beef farm (LORN-F11, Supplementary Table 2), which was included in the study due to its recent history of abortions. The farmer reported rare occurrences of abortions over the past two years, except for a few weeks prior to the sampling, when 2/9 cows aborted. The laboratory results were communicated to the farmer and the veterinarian, who then addressed the cases accordingly.

**Table 1:** Description of the PCR-positive samples and locally circulating pathogenic *Leptospira* spp. isolates obtained from bovine specimens in Austria, November 2021–September 2022.

Isolate ID	Animal ID	Type of sample	Date of sampling (dd.mm.yyyy)	Sampling strategy (place of sampling)	Field inoculation	Time from sampling to inoculation	Ct value^a^	Addition of foetal calf serum^17^	Incubation weeks	Isolation	Dilution which yielded isolation	cgST^b^	cgMLST database ID
21143980-005^c^	LORN-C5	Urine	04.01.2022	Targeted (farm)	No	2:19	26	No	NA	No	NA	NA	NA
21143980-006^c^	LORN-C6	Urine	04.01.2022	Targeted (farm)	No	2:03	25	No	NA	No	NA	NA	NA
22049205-034^c^	LORN-C6	Urine	23.05.2022	Targeted (farm)	Yes	0:03	35	No	NA	No	NA	NA	NA
22049205-037^d^	LORN-C5	Urine	23.05.2022	Targeted (farm)	Yes	0:03	37	No	NA	No	NA	NA	NA
22049205-063	LORN-C249	Kidney	07.07.2022	Targeted (slaughterhouse)	No	3:36	32	Yes	23	Yes	1:1000	1502/1504	1518
22049205-065	LORN-C251	Kidney	07.07.2022	Targeted (slaughterhouse)	No	3:22	34	Yes	18	Yes	1:1000	1502/1504	1521
22049205-068^c^	LORN-C249	Urine	07.07.2022	Targeted (slaughterhouse)	No	3:33	31	No	NA	No	NA	NA	NA
22049205-070^c^	LORN-C251	Urine	07.07.2022	Targeted (slaughterhouse)	No	3:19	33	No	NA	No	NA	NA	NA
22092782-099	LORN-C380	Kidney	22.09.2022	Targeted (slaughterhouse)	No	3:06	33.5	No	7	Yes	1:1000	1504	1520/1519^e^

All isolates were identified as *Leptospira borgpetersenii* serogroup Sejroe, serovar Hardjobovis and belonged to the cluster 40 (clonal group (CG) = 72). Sequencing of the *lfb1* gene confirmed the species *L. borgpetersenii* in five samples. One sample was partially sequenced as *Leptospira.*

^a^PCR targeting the *lipL32* gene.

^b^cgMLST sequence type.

^c^Samples for which the *lfb1* gene could be amplified and subsequently sequenced.

^d^Partial sequence for the *lfb1* gene was obtained.

^e^This isolate was duplicated, one duplicate grew with FCS and the other without FCS. Subsequently, both isolates were characterised.

**Figure 2:**
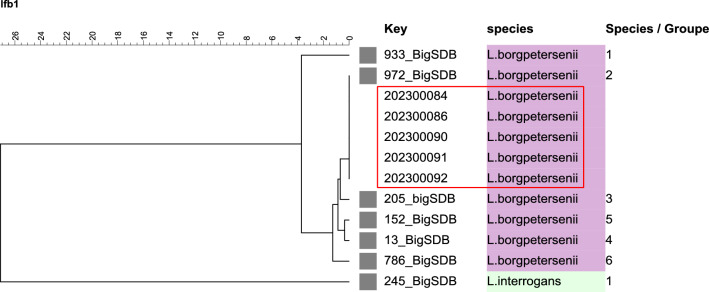
Phylogenetic tree based on *lfb1* sequences showing the position of locally circulating *Leptospira* strains obtained from Austrian bovine samples, Austria, November 2021–September 2022. Maximum likelihood tree inferred from *Leptospira* spp. *lfb1* partial gene polymorphism in cattle samples and reference strains. Grey boxes indicate reference strains, no box indicates the cattle samples (red rectangle).

Sequencing of the *lfb1* gene could be performed on five urine samples (Table 1), all of which were identified as *Leptospira borgpetersenii* species-group (SG) 2, corresponding to serogroup Sejroe serovar Hardjobovis^27^ (Fig. 2). In three samples, amplification of the *lfb1* gene was not successful; in one sample, only partial amplification was achieved.

### Isolation and characterisation of locally circulating pathogenic *Leptospira*

Culture attempts from PCR-positive urine samples, including those inoculated in the field, did not yield successful results (details of the microbiological follow-up of the cultures is provided in Supplementary Table 4). However, we obtained three *Leptospira* isolates from the three PCR-positive kidney samples, representing a success rate of 33% in isolating *Leptospira* from PCR-positive samples. Successful isolation was achieved solely through the 1:1000 dilution. The leptospires exhibited slow growth, showing positive culture after seven (n = 1 isolate), 18 (n = 1), and 23 (n = 1) weeks and requiring the addition of FCS (Table 1). The three isolates were identified as *L. borgpetersenii* serogroup (sg.) Sejroe sv. Hardjobovis and belonged to the cgMLST cluster 40 (CG = 72)^29^ (Table 1), consolidating the results of the *lfb1* gene sequencing.

Note: after deciding to add FCS to support cultures from PCR-positive samples, all were re-inoculated in fresh media with FCS, while the culture without FCS was kept for a few more weeks. Only culture 22092782-099 showed growth in both non-FCS and FCS media. These two clonal isolates were subsequently reported as one isolate.

## Discussion

This study describes the first isolation and subsequent genotyping of zoonotic bacteria of the genus *Leptospira* in Austria. By isolating an autochthonous strain and making it available for the MAT panel, this study marks the initial step towards enhancing the performances of the serological diagnostics for both humans and animals within the country^31^.

The three isolates were identified as *L. borgpetersenii* sg. Sejroe sv. Hardjobovis and belonged to the cgMLST cluster 40 (CG = 72). *Leptospira* strains isolated in 1977 and serotyped as sv. saxkoebing^21^ similarly belong to sg. Sejroe^8^, however, the isolates were not characterised at species level. Cluster 40 includes strains of *L. borgpetersenii* sg. Sejroe sv. Hardjo isolated from cattle and humans in Europe and the USA. Additionally, it contains sv. Hardjobovis strains retrieved from domestic and wild ruminants, as well as humans, in regions spanning New Zealand, Australia, and South America^32^. These findings underscore the widespread geographic distribution of *Leptospira* strains within cluster 40 and emphasise their potential zoonotic risk at the human-ruminant interface^33^.

Although *L. borgpetersenii* sv. Hardjobovis and *L. interrogans* sv. Hardjoprajitno belong to different species, they cannot be differentiated serologically and are both classified as serovar Hardjo^34^. Cattle are recognised as maintenance host of both serovars. Hardjobovis is the most common serovar maintained by cattle worldwide, whereas Hardjoprajitno has been detected in Europe, Africa, South America, but not in the US^35^. Infections by sv. Hardjo have been previously confirmed in humans in Austria^10,36^ and, since 2012, it has led to the hospitalisation of eight persons (including six autochthonous cases), with symptoms ranging from fever and headache to renal failure, meningitis, myalgia, icterus, and exanthem. In three cases, contact with an animal (mouse, livestock, dog) was reported (information source: Austrian Epidemiological Reporting System). Adding an autochthonous Hardjobovis strain to the routine diagnostic MAT panel will definitely improve diagnostic performance for leptospirosis in Austria. Additionally, this holds the potential to provide deeper insights into the epidemiology of the bacteria, particularly concerning its dynamics at cattle-human-environment interfaces, thereby aiding in the selection of appropriate commercial vaccine for both, humans and animals.

Culturing and isolating *Leptospira* from field samples represents a major challenge^17^. In our study isolation could only be achieved using kidney samples and only the dilution 1:1000 generated positive results. Despite our efforts to minimise the time interval between urine sampling and inoculation in culture media, e.g., by optimising the farm-to-laboratory itinerary and implementing in-field inoculation, none of the PCR-positive urine samples yielded positive results in culture. Low culture success rate of *Leptospira* with bovine urine samples has been previously reported^37^. The cell viability of *L. borgpetersenii* sv. Hardjo is negatively affected by prolonged exposure of the strain to urine, especially, an exposure > 2 h significantly reduces the success of isolation^22^. The unsuccessful urine culture may be attributed to the extended time between sample collection and arrival at the laboratory, which exceeded two hours in the case of the PCR-positive urine samples, due to the significant farm-to-laboratory distance. Moreover, the PCR signals observed in the field-inoculated urine samples were weak (Ct values = 35 and 37), suggesting a low bacterial load, which poses challenges for successful culturing. While PCR screening is a valuable tool for prioritising culture follow-up, PCR signal does not provide any information regarding the viability of the bacteria in the sample.

Serovar Hardjo generally shows a fastidious growth and its isolation is difficult, especially due to its requirement for enriched culture medium^17,22^. In this study, the successful culture and isolation of sv. Hardjobovis from bovine samples was optimised by the addition of FCS^17^, despite implementing this procedure at a later stage. This underscores the importance of culture follow-up and the necessity for flexibility in laboratory protocols when handling fastidious-growing strains of *Leptospira*.

Efforts to obtain local isolates are crucial to further our knowledge of the phylogeography and host preference of pathogenic *Leptospira* spp.. Furthermore, the characterisation of *Leptospira* genotypes circulating within a specific region may support the production of locally adapted vaccines. Serovar-specific vaccination can prevent urinary shedding of *Leptospira*^38^, thereby reducing the risk for other animals as well as for individuals at risk of zoonotic occupational exposure.

## Conclusion

We report the first isolation and genotyping of a circulating pathogenic *Leptospira* strain in Austria. Isolating fastidious-growing *L. borgpetersenii* sv. Hardjobovis from a non-endemic region demanded a significant collaborative and transdisciplinary effort, both in the field and in the laboratory. A crucial factor for success relied on the active involvement and cooperation of private veterinarians, farmers, and slaughterhouses^22^. The detection of sv. Hardjobovis on a cattle farm is a notable finding, demonstrating that cattle in Austria may act as carriers of pathogenic *Leptospira,* acting as a possible source of infection of other animals and humans while contributing to environmental contamination through their urine. This information holds significance as leptospirosis is generally regarded as a minor disease in Austria. This study therefore contributes to a better understanding of the local epidemiology of leptospirosis in Austria, and more generally in Europe, and should raise awareness among local stakeholders (particularly farmers, veterinarians, and medical professionals) regarding the zoonotic importance of this pathogen.

### Supplementary Information

Supplementary Information.

Supplementary Tables.

## Data Availability

Data collected in the field as well as microbiological data are available in the Supplementary Materials. The raw data that supports the statistical analysis performed to develop the targeted sampling strategy is available from the Austrian Consumer Health Information System (VIS) and from the Laboratory Information and Management System (LIMS) of the Institute for Veterinary Disease Control Mödling, Austrian Agency for Health and Food Safety (AGES). Restrictions apply to the availability of these data, which were used under license for the current study, and so are not publicly available. Genome sequences generated in this study have been submitted to the *Leptospira* cgMLST database of the Institute Pasteur, and are available publicly at http://bigsdb.pasteur.fr/leptospira, under IDs 1518, 1519, 1520, and 1521.
