# Supplementary material for: First isolation and genotyping of pathogenic Leptospira spp. from Austria
Source: Sci Rep. 2024 Feb 26;14:4467. doi: 10.1038/s41598-024-53775-w (PMC10897423; doi:10.1038/s41598-024-53775-w)
Supplement: Supplementary file 1 — Supplementary Information. [file 41598_2024_53775_MOESM1_ESM.pdf]

Supplementary Material 1. Retrospective analysis of serological data from routine leptospirosis diagnosis in Austrian cattle, 2015-2021

1. Objective

The objective of this analysis was to identify risk factors of bovine leptospirosis in Austria to support the targeted sampling strategy developed in the study.

2. Data preparation

2.1. Laboratory data

Serological results of microscopic agglutination test (MAT) conducted in Austria between January 2015 to 2021 were available. This data originated from the laboratory database of the Austrian Agency for Health and Food Safety (AGES) LIMS. In addition to the serological result, the data included the anonymised ear tag number of the sampled cattle, the sample type, including "routine" sample (taken as part of regular monitoring without suspicion of leptospirosis), "suspect" sample (collected when there is a suspicion of disease), "export" sample (for testing cattle intended for export), or "private" sample (taken outside the regular monitoring without suspicion of leptospirosis), and the entry date.
Each sample was tested for eight serogroups/serovars of *Leptospira* (Hardjo, Saxkoebing, Australis, Canicola, Icterohaemorrhagiae, Grippotyphosa, Pomona, Tarassovi); some individual animals were investigated more than once during the study period. During the data processing phase, the 78,319 serological results were consolidated into one result per sample. A sample was considered positive if reaction with a titre ≥ 100 against at least one serotype was detected. This resulted in 13,754 serological results from 8,839 cattle, which were used for further analysis.

2.2. Data from the Consumer Health Information System (VIS) and data processing

Data on cattle, cattle movements as well as farm activity and location were extracted from the Austrian Consumer Health Information System (Verbrauchergesundheitsinformationssystem, VIS), where a large amount of official information relevant to veterinary and food safety is stored. The different datasets were merged via farm ID and/or via the animal ear tag number. The farm ID of both, the sampling location and the farm, the ear tag number and the ID of the sample were anonymised before the statistical analysis of the data.

The farms of the sampled cattle were determined by cross-referencing the ear tag number and the entry date in the laboratory dataset in combination with the cattle movement data. Subsequently, the farm of the sampled cattle was identified as the farm where the animal had resided for a minimum of 30 days prior to sampling. In total, the dataset contained data from 3,040 farms, including semen collection centres.

For each animal, the following data were extracted using the ear tag number: date of birth, date of death, breed, sex, whether the animal had calved or not, and whether it had been on a community pasture or pasture at least once. Additionally, the number of times and days a cattle has been on a (community) pasture was calculated. A total of 1,366 cattle were brought to (community) pastures during the study period (Table S1).

Table S1: Data extracted from the VIS database, including potential risk factors of leptospirosis at animal level.

Variable	Description
Cattle ID	Cattle ear tag number (anonymised)
Breed	Cattle breed
Sex	Sex of the cattle
Age	Age in days or years
Calved	Calved yes/no
Community pasture	Cattle were on a community pasture (definition: more than one farm brought animals to this alp in one year) yes/no
Number of days on a pasture (generally and analogously for community pastures)	Number of days on a pasture as of 2010 (a standardised value was used in the analysis, calculated as the proportion of cattle's life years on an alpine pasture)
Number of stays on a pasture (generally and analogously for community pasture)	Number of stays on a pasture as of 2010 (a standardised value was used in the analysis, calculated as the proportion of cattle's life years on an alpine pasture)

For each farm, the following data was extracted: averaged number of cattle, pig, sheep, and goat stocks from 2015 to 2020. Animal movements were considered from 2010 onwards. For each farm, the average number of cattle received from within the country was calculated per year, as well as the average number of animals received from abroad. The number of domestic farms from which arrivals came in each year was also averaged, as was the number of foreign farms from which arrivals came to the respective farm (Table S2).

Table S2: Data extracted from the VIS database, including potential risk factors of leptospirosis at farm level.

Variable	Description
Farm ID (farms)	Farm where the animal was last kept for at least 30 days prior to the sampling date (anonymised).
Federal state (farms)	Federal state of the farms.
Stock cattle	Average annual stock of cattle on the farm since 2015 (as of the reporting date of April 1st each year).
Stock pigs	The cattle farm is also an active pig farm yes/no (the number of pigs per year since 2015 as of the reporting date of April 1st was considered)
Stock sheep	The cattle farm is also an active sheep farmer yes/no (the number of sheep per year since 2015 as of the reporting date April 1st was considered).
Stock goats	The cattle farm is also an active goat farmer yes/no (the number of goats per year since 2015 as of the reporting date April 1st was considered).
Stock cows	Mean annual number of cows on the farm since 2015 as of the reporting date April 1st.
Type of farm milk	Farm has farm type "milk delivery or direct marketing" (yes/no).
Number of domestic movements (cattle)	Number of annual cattle introductions to the herd from within Austria as of 2010 (averaged).
Number of movements abroad (cattle)	Number of annual cattle introductions to the herd from abroad as of 2010 (averaged)
Number of domestic movements (farms)	Number of farms located in Austria from which cattle were introduced to the herd (from 2010, averaged)
Number of movements abroad (farms)	Number of farms located abroad from which cattle were introduced to the herd (from 2010, averaged)

All samples from cattle collected at semen collection centres were excluded from the analysis and only complete data sets were included, ensuring that all necessary information for each case was available. Animals which tested positive at least once during the study period were considered "leptospirosis positive". If only negative test results were available, the animal was considered "negative". The resulting dataset included 8,431 sampled animals from 3,030 herd farms. More than 100 animals were sampled from six farms only (mostly export samples), while only one sample result was available from 1,498 other farms.

3. Data situation

In total, 441 out of the 8,431 sampled animals tested positive for leptospirosis. This corresponds to a proportion of 5.23% (apparent prevalence) (95% CI: 4.77–5.73). This proportion varied greatly depending on the sample type (Table S3).

Table S3: Proportion of positive samples (incl. 95% confidence interval), average age, proportion of cows that have calved and proportion of male cattle per sample type.

	Export samples	Private samples	Routine samples	Suspect samples
**Sample size**	5,767	1,601	934	129
**Proportion of positive [95% CI]**	2.3% [2.0–2.8]	12.3% [10.7–14.0]	6.4% [4.9–8.2]	36.4% [28.1–45.4]
**Mean age (years)**	1.6	3.6	4.0	5.2
**Proportion of cows which have calved**	1.2%	56.7%	69.2%	96.9%
**Proportion of males**	4.7%	34.9%	27.9%	0.0%

The origin of the samples also varied per sample type. Most of the "export" samples came from Upper and Lower Austria (49.4% and 23.9% of all "export" samples respectively), while most "suspect" samples came from the Tyrol/Vorarlberg region (73.6% of "suspect" samples). Regarding the entry date of the samples, very few "export" samples were recorded in 2016 (2.9% of the samples from 2016), although these clearly predominated overall (between 46.7 and 87.9% annually).

The descriptive analysis revealed the presence of distinct subsamples in the data depending on the type of sample, as the likelihood of a cattle belonging to a particular subsample depends on characteristics such as age, region, sex, and health condition. For example, the data suggested that export cattle were primarily young, male, and apparently healthy animals, where the suspect samples were from older animals with abnormal health. Given

the imbalance in the sample distribution, it was deemed appropriate to conduct separate analyses for these subsamples in addition to the overall analysis.

4. Statistical analysis

The probability of a positive sample result for leptospirosis in a cattle π_i is modelled using a Generalised Linear Mixed Model (GLMM) as a function of explanatory variables $\boldsymbol{x_i} = (x_{i1}, \dots, x_{ip})$ and a random effect per farm u_j. Let y_i be the binary test result for cattle i. The logistic model is thus given as $y_i|u_j \sim Bernoulli(\pi_{ij})$, with $Logit(\pi_{ij}) = \beta_0 + \boldsymbol{x_i\beta} + u_j$ with u_j~N$(0, \tau^2)$.
Model and variable selection were performed by forward selection using the Bayesian Information Criterion (BIC) and a 5-fold cross validation (CV) using the Matthews correlation coefficient (MCC). For the calculation of the MCC, the optimal cut-off for the prediction was determined for each model using the ROC curve.
The statistical modelling was implemented in the statistical software R 4.0.2 [1] with the help of the packages glmmTMB [2] and mltools [3]. The package binGroup [4] was used to calculate confidence intervals.

5. Results

The results of the model selection are shown for the (sub)samples in Table S4. All models showed that variables related to alpine farming (stays or number of days on alpine pastures or community alps) significantly increased the risk of leptospirosis. The variable having calved ("calved") also consistently had a significant positive impact on the probability of a positive sample.

Table S4: Result of the variable selection (final models per subsample)

Model selection	Variable	BIC	CV MCC
**Export samples (n=5,676, 2,013 farms)**			
**BIC**	Use of alpine pastures (yes/no)	1,081	0.11
**CV**	Number of stays on a community alp (standardised)	1,083	0.14
**Private samples, routine samples, suspect samples (n=2,664, 1,182 farms)**			
**BIC**	Sample type	1,569	0.20
**CV**	Number of days on a community alp (categorised) + year	1,608	0.28
**Private samples, routine samples (n=2,535, 1,125 farms)**			
**BIC**	Sample Type + Calved	1,405	0.21
**CV**	Number of stays on a community alp (standardised) + calved	1,408	0.24
**Total sample (n=8,431, 3,030 farms)**			
**BIC**	Calved + number of stays on a community alp (standardised)	2,680	0.27
**CV**	Number of stays on a community alp + year + calved	2,711	0.31

The detailed results for the subsample of “private” and “routine” samples determined using the model selection criterion BIC and, for the total sample, determined via cross validation (MCC), are presented below (Table S5 and Table S6). According to this, in the “private” and “routine” data, “routine” samples had 0.20 times (corresponds to $\exp(-1.62)$) likelihood of a positive result compared to private samples. Cattle that had not (yet) calved also had a significantly lower risk (0.23 times likelihood) of testing positive compared to cattle that had already calved.

Table S5: Model results estimated from private and routine samples (model selection using BIC).

**Variable**		**Estimated parameter $\widehat{\boldsymbol{\beta}}$**	**p-value**
**Intercept**		-7.58	
**Sample type**	Private (reference)		
	Routine	-1.62	0.006
**Calved**	Yes (reference)		
	No	-1.46	0.020
**Variance component farm $\widehat{\boldsymbol{\tau^2}}$**		112.80	

Table S6: Model results estimated from the total sample (model selection using cross validation).

**Variable**		**Estimated parameter $\widehat{\boldsymbol{\beta}}$**	**p-value**
**Intercept**		-7.07	<0.001
**Number of stays on a community alp**		0.20	0.004
**Year**	2015 (reference)		
	2016	0.56	0.170
	2017	-0.40	0.275

	2018	-0.84	0.044
	2019	-1.31	0.007
	2020	-1.20	0.003
	2021	-1.86	0.002
**Calved**	Yes (reference)		
	No	-1.53	<0.001
**Variance component farm** $\widehat{\tau^2}$		81.49	

6. Discussion and Summary

When all sample types are modelled together, it is apparent that sample type or variables that vary greatly per sample type have a large impact on the probability of leptospirosis. This can be explained by the different observed apparent prevalence in the subsamples and their different composition. The reason for this lies in the biased sample. The sampled animals were not representative for the cattle population in Austria. Whereas young, apparently healthy, male cattle that were aimed at export were overrepresented due to the large number of export samples, young, symptomatic, and healthy, older animals were less likely to be included in the sample (for example, these were exported less frequently and were, therefore, not tested routinely for export purpose). Some potential influencing variables were correlated. For example, the variable "calved" is naturally correlated with the age and sex of the cattle, as well as with the sample type. Also, therefore, the result of the statistical analysis does not mean that those variables that were not included in the models (such as age of cattle) have no influence on the probability of leptospirosis. However, the influence of the identified variables (e.g. “calved”) is stronger. Because of the stepwise forward selection of explanatory variables, the influential variable with the highest explanatory power is included in the model at each step, and explanatory variables correlated with it often do not provide any additional relevant explanatory power.

The low values for the MCC (0.11 to 0.31) generally indicate a relatively low explanatory power for the models. Values close to one would mean that both negative and positive samples are almost perfectly explained by the model. Nevertheless, across all subsamples and models, grazing on community pastures and the variable "calved" were identified as risk factors.

7. References

1. R Core Team R. A language and environment for statistical computing. R Foundation for Statistical Computing https://www.R-project.org/ (2020).
2. Brooks, M.E. *et al.* glmmTMB balances speed and flexibility among packages for zero-inflated Generalized Linear Mixed Modeling. *The R Journal* **9,** 378–400; 10.32614/RJ-2017-066 (2017).
3. Gorman, B. mltools: machine learning tools https://CRAN.R-project.org/package=mltools (2018).
4. Zhang, B., Bilder, C., Biggerstaff, B., Schaarschmidt, F., Hitt, B. binGroup: evaluation and experimental design for Binomial Group Testing https://CRAN.R-project.org/package=binGroup (2018).
